# Treatment of Refractory Ischemic Priapism: A Case Report and Literature Review

**DOI:** 10.7759/cureus.39882

**Published:** 2023-06-02

**Authors:** Jose Rogelio Vazquez Gonzalez, Roberto Cortez Betancourt, Jose Gerardo Alvarez Lopez, David Cortez Ramirez, Oscar Uriel Garcia Rivera

**Affiliations:** 1 Urology, Centro Medico Nacional 20 de Noviembre, Instituto de Seguridad y Servicios Sociales de los Trabajadores del Estado, Mexico City, MEX

**Keywords:** surgical treatment of ischemic priapism, medical treatment of ischemic priapism, erectile dysfunction, high flow, low flow, priapism refractory to treatment, ischemic priapism, priapism, recurrent priapism, stuttering priapism

## Abstract

Recurrent priapism is a rare and poorly known entity. It is defined by recurrent episodes of painful erections that last less than four hours. The etiology is similar to that of ischemic priapism. Episodes lasting more than four hours require immediate intervention to prevent penile fibrosis and subsequent erectile dysfunction.

A 42-year-old male with no significant chronic-degenerative history was referred to our medical center from his second-level medical unit after a 56-hour history of ischemic priapism with the persistence of tumescence despite medical and surgical treatment. Upon interrogation, the patient reported stuttering (recurrent) episodes of painful erections lasting approximately three to four hours, not associated with sexual activity or arousal, in the past two years, with spontaneous resolution. He denied the use of psychotropics or drugs for erectile dysfunction. As a palliative measure, a left saphenous-cavernous (Grayhack) bypass was performed, with a 90% decrease in tumescence and total resolution of pain during the first 12 hours.

There is little information and treatment recommendations for patients with recurrent priapism, and even less for patients who are refractory to conventional medical and surgical treatment. Recurrent or stuttering priapism is a condition with a low incidence and a pathophysiology compatible with low-flow priapism. It is difficult to treat and has a poor prognosis in terms of erectile function. Likewise, it is mostly associated with the use of psychotropic drugs such as cocaine and marijuana, medications for erectile dysfunction such as phosphodiesterase inhibitors, prostaglandin E1 analogues, and hematological malignancies such as sickle cell anemia and multiple myeloma. The aim of this article is to share our experience with a patient refractory to multiple medical and surgical treatments.

## Introduction

The word “priapism” originated in Greek mythology. Priapus was the god of fertility, and his large phallus was the symbol of masculine power [1]. The first case of priapism was published by Callos in 1845 [2]. Priapism is a sexual dysfunction in which there is a prolonged, persistent, and painful erection of the penis without stimulation or desire for more than four hours [3]. It is considered a urologic emergency due to the possibility of ischemia, necrosis, compartmental syndrome, and fibrotic lesions of the cavernous erectile tissue, as well as irreversible changes in the cavernous and endothelial smooth muscle cells [4]. The etiological and pathophysiological process of priapism is still poorly understood. From the hemodynamic point of view, by measuring intracavernous blood gases, priapism can be classified into two types: ischemic (veno-occlusive, low flow) and non-ischemic (arterial, high flow) [4].

According to the American Urological Association (AUA) guidelines, priapism can occur in the following forms: acute (usually low flow), chronic (usually high flow), and intermittent (recurrent or stuttering, usually ischemic) [5,6]. The continuous, prolonged, recurrent, and damaging changes are due to multiple etiologies that can result in irreversible erectile dysfunction (ED). Current practices lack an understanding of its pathophysiology, especially at the molecular level. Traditional treatments are empirical, without methodology and a strict protocol to follow. The results regarding the return to normal erectile function are poor, especially in cases of recurrent priapism. Therefore, it is essential to understand the molecular mechanisms for the formulation of rational, strategic, and preventive treatments for high-risk populations, such as patients with sickle cell anemia and recurrent priapism [7].

## Case presentation

A 42-year-old male with no significant chronic-degenerative history was referred to our medical center from his second-level medical unit after a 56-hour history of ischemic priapism. The patient was admitted to the emergency department of his second-level unit for presenting a painful and persistent five-hour erection. A corpora cavernosa blood gas test was performed, obtaining the following results: potential of hydrogen (pH) 6.98, partial pressure of oxygen (pO2) 34%, partial pressure of carbon dioxide (pCO2) 82% (reference values for ischemic and non-ischemic priapism, and normal mixed blood are described in Table 1).

**Table 1:** Typical blood gas reference values These values are as per the guidelines of the AUA [8]. pH: Potential of hydrogen, pO2: Partial pressure of oxygen, pCO2: Partial pressure of carbon dioxide, AUA: American Urological Association

Source	pH	p02	pC02
Normal arterial blood	7.4	>90	<40
Acute ischemic priapism	<7.25	<30	>60
Normal mixed venous blood	7.35	40	50

Diagnosis of ischemic/low-flow priapism was integrated. Physicians proceeded to perform lavage and aspiration of the corpora cavernosa with phenylephrine every three to five minutes for a period of one hour, with a partial decrease in tumescence for approximately two hours. The patient subsequently presented a recurrence of tumescence and pain, for which he was admitted to the operating room. A Winter-type distal cavernotomy was carried out, but no remission or decrease in tumescence was achieved. Then an Al Ghorab-type open distal bypass was performed and a decrease in tumescence of approximately 40% was obtained for a period of 48 hours, after which tumescence and pain increased. The patient was once again rushed to the operating room and underwent a spongiosum-cavernous fistula (Quackels technique). Since a decrease in tumescence was not achieved and due to persistent pain, the patient was referred to our third-level care center to continue his management. Upon admission, the patient was found with a painful erection on palpation, with total tumescence and rigidity of both corpora cavernosa, with injuries to the glans and base of the penis corresponding to the procedures performed in the referral medical unit. A penile Doppler ultrasound was carried out to find decreased cavernous arterial flow compatible with persistent ischemic priapism. The toxicological and hematological panel was negative.

Upon interrogation, the patient described approximately seven episodes of spontaneous and painful morning erections lasting approximately three to four hours throughout the last two years, with spontaneous resolution and normal sex life with a previous International Index of Erectile Function (IIEF-5) score of 23 points (21-25 normal erectile function). He denied the use of psychotropic drugs or drugs for erectile dysfunction, and he was not under any kind of medication nor had any known hematologic malignancies.

We then discussed with the patient that the total loss of erectile function was irreversible, secondary to the time of evolution and all the surgical procedures performed, so the placement of a penile prosthesis was recommended. However, the public health institution does not have the required resources and penile prostheses are not covered by public health programs. Therefore, the patient was admitted to the operating room, and a left cavernous-saphenous shunt (Grayhack technique) was performed. A 90% decrease in tumescence during the first 12 hours and a total resolution of pain were achieved. During follow-up, the patient reported an absence of pain, but also a total absence of erections with an IIEF-5 score of 0 points (0-7 severe erectile dysfunction).

Description of the Grayhack surgical technique

**Figure 1:**
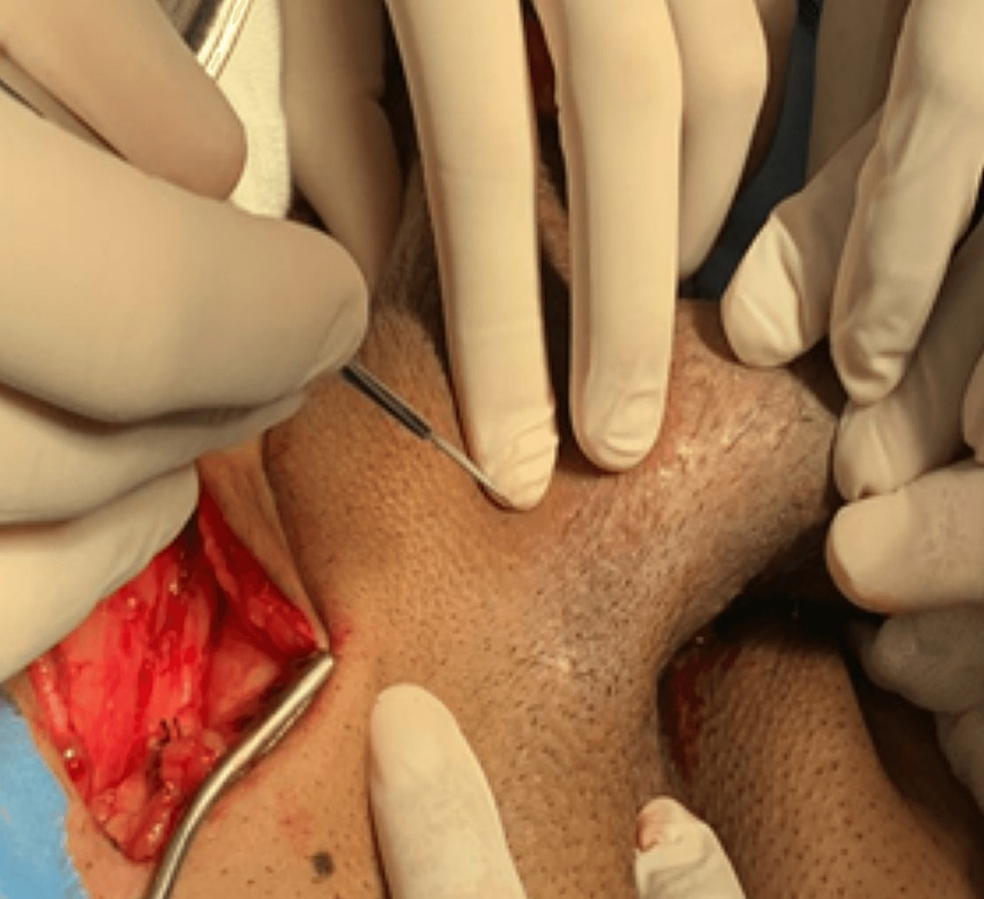
An incision is made on the skin to reach the base of the left corpus cavernosum

Approximately 15 cm of the left superficial saphenous vein was procured and flushed/irrigated with a 14 Fr Jelco catheter using 20 ml of saline solution mixed with 5000 IU of unfractionated heparin. An incision was then made on the skin to reach the base of the left corpus cavernosum (Figure 1).

**Figure 2:**
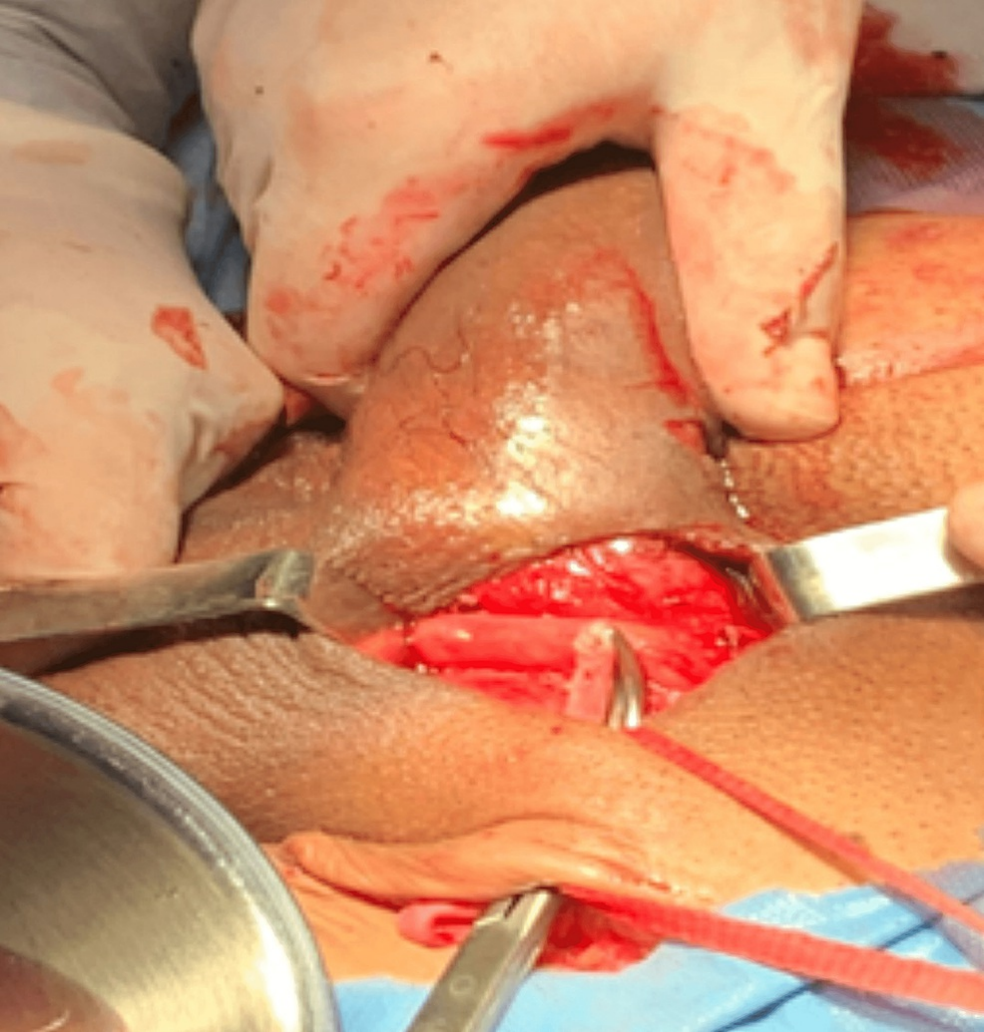
Subcutaneous tunnel and saphenous vein positioning

A superficial subcutaneous tunnel was made from the inguinal region to the base of the corpus cavernosum. The saphenous vein was carefully positioned to avoid torsion or strangulation (Figure 2).

**Figure 3:**
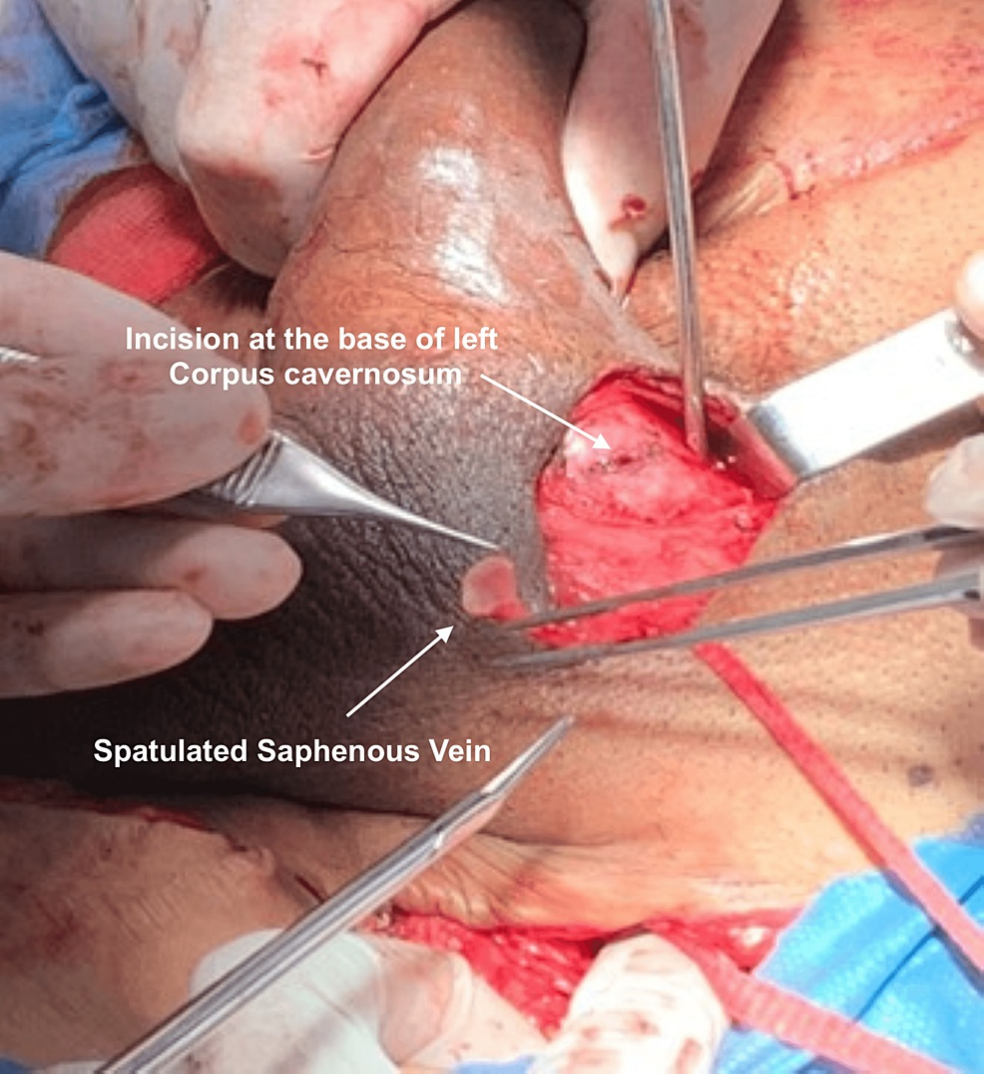
Incision in the corpus cavernosum and spatulation of the saphenous vein

Then, an incision was made in the corpus cavernosum to spatulate the saphenous vein (Figure 3).

**Figure 4:**
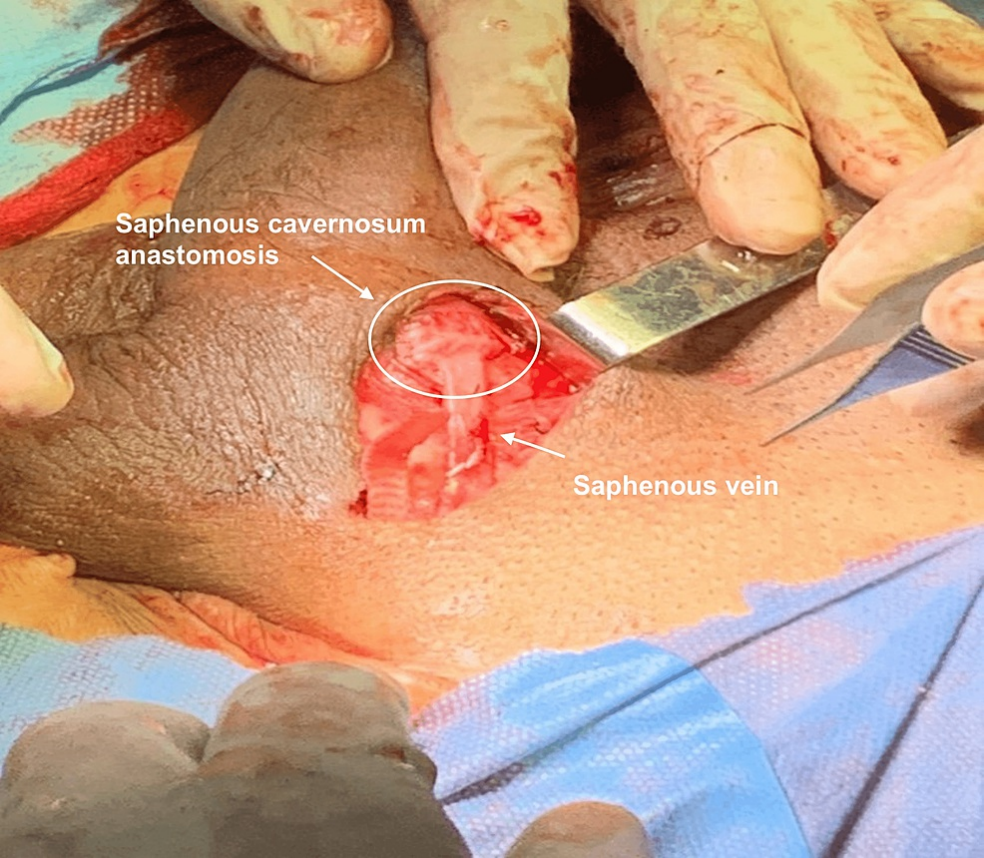
Saphenous vein anastomosed to left corpus cavernosum

**Figure 5:**
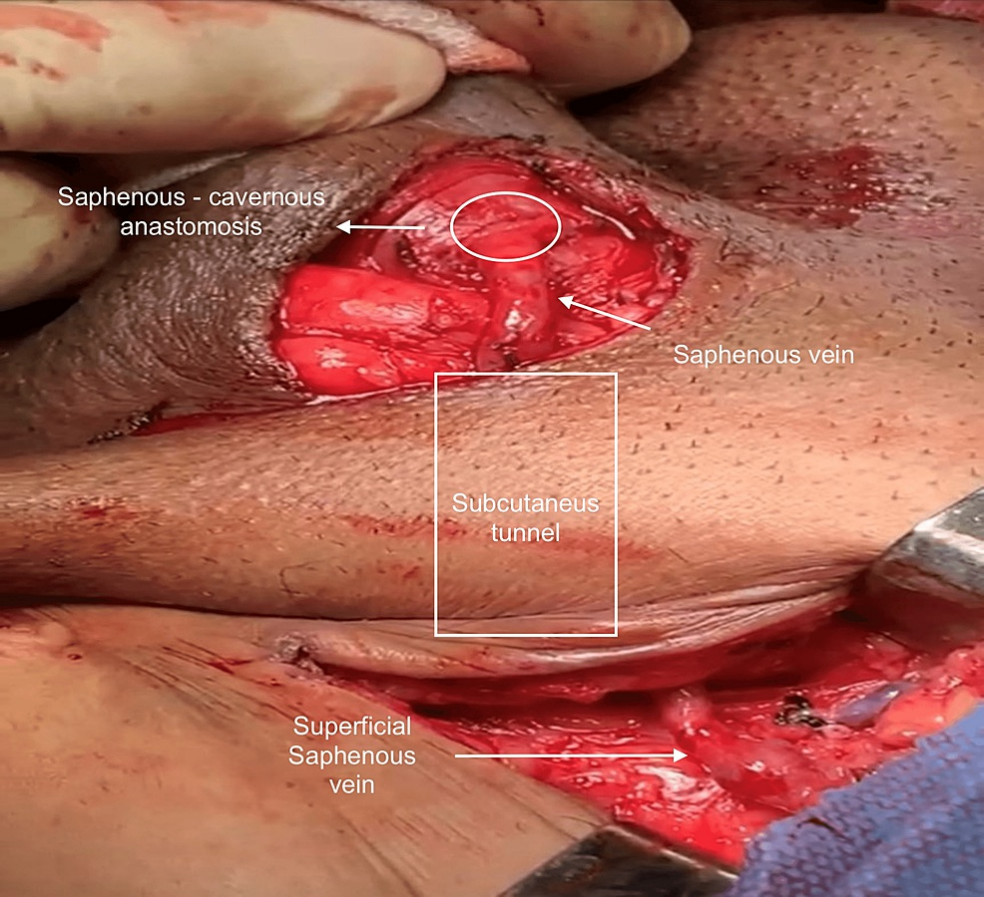
Intraoperative image of saphenous vein anastomosis to left corpus cavernosum

The superficial saphenous vein was anastomosed to the left corpus cavernosum with polydioxanone suture (PDS) 5-0 simple stitches (Figures 4-5).

During the procedure and immediate postoperative period, 5000 IU of unfractionated heparin was administered, followed by an anticoagulation regimen based on low molecular weight heparin (enoxaparin) 80 mg subcutaneous (SC) for seven days, followed by factor Xa inhibitor (rivaroxaban) 20 mg orally once a day for four months, to prevent thrombosis of the saphenous-cavernous shunt.

## Discussion

Mechanism of ischemic priapism

Ischemic priapism consists of a dysfunction between the relaxation and constriction mechanisms, which predispose the penis to hypoxia and acidosis conditions [3]. According to experimental studies, irreversible damage to the penis is known to occur after 12 hours of having this type of priapism [6]. The mechanical type is associated with an 80% occurrence related to multiple blood flow disorders and refers to the etiology of thromboembolic phenomena, hyperviscosity syndromes as seen in acute leukemias, and congenital hemoglobin abnormalities that cause sickle cell anemia, which is common in the Black population [4]. There are other causes associated with this clinical condition, such as pelvic abscesses; penile tumors; perianal tumors; the use of drugs such as trazodone, marijuana, cocaine, and alcohol; and intracavernous injections of vasoactive agents. The nervous type present in 20% of cases refers to suspicions of neurological disorders that supposedly affect the center of the erection (20% of cases of priapism are suspected to be caused by neurological disorders that affect the center of the erection). This category includes infections such as syphilis, brain tumors, epilepsy, poisoning, brain injuries, and spinal cord trauma [9].

Diagnosis

Diagnosis is performed by integrating clinical history, including duration, the presence and/or absence of pain, and corpus cavernosum gasometry. Within the clinical context of ischemic or low-flow priapism, rigid corpora cavernosa, pain, and a corpus cavernosum blood gas showing hypoxia, hypercapnia, and acidosis can be found (Tables 1-2).

**Table 2:** Key findings in priapism evaluation These findings are consistent with the information provided in the guidelines of the AUA. However, these are of personal authorship and are different from those published in the guidelines [8]. AUA: American Urological Association

Finding	Non-ischemic priapism	Ischemic priapism
Penile pain	Absent	Present
Corpora cavernosa fully rigid	Absent	Present
Abnormal cavernous blood gases	Absent	Present
Hematologic malignancies	Absent	Sometimes present
Chronic well-tolerated tumescence without full rigidity	Present	Absent
Recent intracavernous vasoactive drug injection	Absent	Sometimes present
Perineal trauma	Sometimes present	Absent

Pharmacological treatment of priapism

For non-ischemic priapism, conservative management is the first line of treatment; therefore, selective embolization and surgeries are reserved for refractory cases and severe trauma. In contrast, ischemic priapism requires immediate intervention, such as suction and irrigation, to resolve the established compartment syndrome [5,9].

The first part of the treatment must be the aspiration and irrigation of the cavernous bodies using a Jelco 19 or 21F and the extraction of 5 cm3 to 20 cm3 of blood with three objectives: 1) to confirm diagnosis via gasometry analysis for hypoxemia, hypercapnia, and acidosis, especially as dark blood is produced in this form of priapism; 2) to extract blood to reduce pain; and 3) to aspirate to cause a contraction of the smooth muscle of the penis, which resolves the event of priapism [5].

Intracavernous application of sympathomimetics is still the best option if the condition persists. The agent of choice is phenylephrine in a 100 to 500 µg/ml dilution and this should be done in repeated intracavernous injections every five minutes for a maximum of one hour. Finally, if necessary, surgical treatment with shunts is indicated [9]. The goal is to quickly resolve the unwanted erection condition and prevent damage to the cavernous erectile tissue. Although management in cases of intermittent priapism is under development, current pharmacological therapies include antiandrogens, baclofen, ketoconazole, digoxin, methylene blue, and terbutaline, among others, which have little efficacy or fail to address the neurotransmission mechanisms in penile vascularization [5,10]. The AUA only supports the use of hormonal treatment as first-line therapy; however, this brings sexual and systemic complications due to androgen deficiency [5,9]. Molecular studies are underway with the continuous need for new therapies and natural preventive measures of practical application that demonstrate a decrease in recurrent priapism episodes, especially in the high-risk population (sickle cell anemia, use of psychotropic drugs, and pharmacological therapies for ED). Recent evidence shows that priapism is related to the dysregulation of penile phosphodiesterase 5 (PDE5) expression [11]. Phosphodiesterase 5 is a key molecule for regulating the erectile response and has a marked influence on vascular tone. Studies have shown that long-term use of PDE5 inhibitors can correct or restore enzyme activity and thus prevent the recurrence of priapism episodes [12].

Champion et al. established an experimental model of priapism in nitric oxide-deficient rats, where this phenotype was related to penile PDE5 dysregulation (nitrate reserve tolerance). The chronic use of sildenafil in these phenotypes resulted in penile PDE5 regulation and, subsequently, a lower incidence of priapism episodes [11]. Burnett et al. administered sildenafil 25 mg daily and compared it to tadalafil 5 mg three times a week in a series of men with sickle cell disease and found that priapism episodes decreased in both groups over the long term [12]. Low-dose PDE5 inhibitor therapy has become a paradoxical treatment for priapism as it is similar to the medication normally prescribed to improve erections. However, while PDE5 inhibitors show signs of efficacy in preventing recurrent priapism, additional conditions still need to be studied [12]. Another experimental study demonstrated the role of transforming growth factor (TGF)-beta 1, a cytokine that is vital for tissue repair and is involved in the progression of fibrosis that occurs in priapism. Regulation of TGF-beta1 occurs during the period of hypoxia and in response to oxidative stress [12].

Researchers recently demonstrated that early administration of TGF-beta1 neutralized the action of antibodies in mice, limiting fibrotic action. Finally, the evidence shows that both in animal and human models, the PDE5 inhibitor acts as a regulator of nitric oxide, thus indicating the reduction of complications such as fibrosis and restoring the physiological conditions of the penis [13].

Surgical treatment of ischemic priapism

When all conservative and pharmacological options have been exhausted, surgical management can be used as a second line of treatment for refractory priapism. However, surgical treatment of priapism is unlikely to preserve erectile function and is a more palliative measure to alleviate pain [14]. Most of the time, when surgical management is considered, the erectile function is already significantly damaged, with little chance of returning to normal [14].

Almost all guidelines and urology surgical books recommend a percutaneous distal shunt (corporoglanular) as the first surgical procedure, so medical specialists must be familiar with the three most common percutaneous distal shunts (Winters, Ebbehoj, and T-Shunt). They all consist of creating a window (fistula) between the tunica albuginea and the corpus cavernosum [14,15].

Our center recommends that the first attempt be the Winters procedure because it is less invasive and there is a greater possibility of regaining erectile function if extensive fibrosis of the corpus cavernosum has not yet been generated. When preserving the erectile function is no longer of interest in relation to the time elapsed with the priapism (>36 hours), then an Al-Ghorab open shunt (opening of the corpus cavernosum) or Burnett procedure (opening and canulating the corpus cavernosum) must be assessed to generate much greater communication between the ischemic corpus cavernosum and the corpus spongiosum, thus increasing the probability of success but definitively damaging the corpus cavernosum to a point of no return. If none of the above percutaneous or open distal shunts are successful in obtaining detumescence, the next option is a proximal shunt like the Quackels technique, in which a corporo-spongiosal fistula is created [14,15].

Two other important and common procedures involve the creation of a vein anastomosis shunt. They are used as an alternative treatment for the resolution of refractory priapism. One of them is the Grayhack technique, which involves a saphenous vein-to-corpus cavernosum anastomosis, thus creating a unilateral or bilateral caverno-saphenous shunt. We used this technique on our patient. The last known technique is the Barry caverno-dorsal vein anastomosis [14,15].

Anticoagulation

Current literature recommends prophylaxis with aspirin, clopidogrel, or both [15] to prevent the shunt from clotting, but there is no consensus about a regimen or for how long it should be used. Our center used 5000 units of unfractionated heparin during the procedure and another dose in the immediate postoperative period. Subsequently, we used low molecular weight heparin (enoxaparin, full anticoagulation scheme) for seven days and discharged the patient home with factor Xa inhibitor (rivaroxaban) 20 mg once a day for four months, with excellent outcomes.

## Conclusions

Although priapism is a rare occurrence, affecting only a small portion of the male population, early diagnosis, and correct management are necessary to prevent complications such as irreversible ED and compartment syndrome. An understanding of the molecular mechanisms involved in the pathogenesis will bring greater advances in future medical interventions. Currently, drugs that act on the nitric oxide/cyclic guanosine monophosphate/PDE5 cascade and prevent the release of cytokines are on the therapeutic investigation list.

With this literature review and the presentation of the clinical case, we seek to share the experience of the surgical technique and results of the saphenous-cavernous shunt, as well as the anticoagulation regimen used to prevent thrombosis of the shunt in a patient with priapism refractory to multiple medical and surgical treatments.
